# Direct preparation of solid carbon dots by pyrolysis of collagen waste and their applications in fluorescent sensing and imaging

**DOI:** 10.3389/fchem.2022.1006389

**Published:** 2022-09-12

**Authors:** Xiaoyun Qin, Cuicui Fu, Jin Zhang, Wenlong Shao, Xiaomei Qin, Yanghai Gui, Lan Wang, Huishi Guo, Fenghua Chen, Liying Jiang, Gang Wu, Floris J. Bikker, Dan Luo

**Affiliations:** ^1^ School of Material and Chemical Engineering, Zhengzhou University of Light Industry, Zhengzhou, China; ^2^ CAS Center for Excellence in Nanoscience, Beijing Key Laboratory of Micro-nano Energy and Sensor, Beijing Institute of Nanoenergy and Nanosystems, Chinese Academy of Sciences, Beijing, China; ^3^ Department of Oral Biochemistry, Academic Centre for Dentistry Amsterdam (ACTA), University of Amsterdam (UvA) and Vrije Universiteit Amsterdam (VU), Amsterdam, Netherlands; ^4^ School of Electrical and Information Engineering, Zhengzhou University of Light Industry, Zhengzhou, China; ^5^ Department of Oral and Maxillofacial Surgery/Pathology, Amsterdam UMC and Academic Center for Dentistry Amsterdam (ACTA), Amsterdam Movement Science, Vrije Universiteit Amsterdam, Amsterdam, Netherlands; ^6^ Department of Oral Cell Biology, Academic Center for Dentistry Amsterdam (ACTA), University of Amsterdam and Vrije Universiteit Amsterdam, Amsterdam, Netherlands

**Keywords:** carbon dots, biomass, conversion yield, sensing, iron ions, bioimaging

## Abstract

The fluorescent carbon dots (CDs) have found their extensive applications in sensing, bioimaging, and photoelectronic devices. In general terms, the synthesis of CDs is straight-forward, though their subsequent purification can be laborious. Therefore, there is a need for easier ways to generate solid CDs with a high conversion yield. Herein, we used collagen waste as a carbon source in producing solid CDs through a calcination procedure without additional chemical decomposition treatment of the raw material. Considering a mass of acid has destroyed the original protein macromolecules into the assembled structure with amino acids and peptide chains in the commercial extraction procedure of collagen product. The residual tissues were assembled with weak intermolecular interactions, which would easily undergo dehydration, polymerization, and carbonization during the heat treatment to produce solid CDs directly. The calcination parameters were surveyed to give the highest conversion yield at 78%, which occurred at 300°C for 2 h. N and S atomic doping CDs (N-CDs and S-CDs) were synthesized at a similar process except for immersion of the collagen waste in sulfuric acid or nitric acid in advance. Further experiments suggested the prepared CDs can serve as an excellent sensor platform for Fe^3+^ in an acid medium with high anti-interference. The cytotoxicity assays confirmed the biosafety and biocompatibility of the CDs, suggesting potential applications in bioimaging. This work provides a new avenue for preparing solid CDs with high conversion yield.

## Introduction

With high photostability, low cytotoxicity, easy surface modification, and chemical inertness, carbon dots (CDs) have been an important member of nanocarbon family that includes nanodiamond, graphene, graphdiyne, carbon nanotube, and fullerene. Hitherto, the CDs have found their applications in *e.g.* bioimaging, drug delivery, fluorescence sensing, photocatalysis, and multicolor light-emitting diodes (LEDs) (Wareing et al., 2021). Since the first discovery of CDs by Xu and co-workers in 2004 (Xu et al., 2004), various precursor carbon sources and preparation methods have been exploited to achieve the production of these nanocarbon structures. In general terms, the preparation of CDs can be either top-down or bottom-up method, where the first approach depends on some equipment or environment to supply harsh conditions (laser, arc discharge, strong acid, *etc.*) in exfoliating CDs from bulk graphite-like carbon sources. In contrast, the bottom-up approach usually involves facile heat treatment to achieve pyrolysis and carbonization from cheap carbon precursors, such as glucose, ethylenediamine, urea, and various biomasses. Various thermopolymerization approaches including solvothermal, microwave, and reflux are frequently used in the preparation of CDs. In line, the authors have also previously used pomelo peel, willow bark, flour, and collagen scaffolds as carbon sources to produce CDs by hydrothermal processing (Lu et al., 2012; Qin et al., 2013a; Qin et al., 2013b; Qiao et al., 2018). Also, biomass waste has been extensively surveyed in the fabrication of CDs on a large scale from the point of view of economy and waste utilization.

However, necessary subsequent purification and solidification procedures are generally laborious, leading to low yields, and difficulties in large-scale production procedures. To exemplify, Park and co-workers transformed 100 kg of food wastes into ∼120 g CDs (with the conversion yield of 0.12%) by microwave radiation of the food wastes/ethanol solution for 45 min, while centrifugation and filtration treatments were carried out to remove large particles (Park et al., 2014). Others already found ways to improve the efficiency and demonstrated a one-step pyrolytic treatment of chia seeds using a muffle furnace at 350°C for 6 h to achieve CDs with a yield of ∼10% (Jones et al., 2017). Moreover, Yang and co-workers showed a strong improvement in efficiency as they reported a top-down method to exfoliate CDs from Chinese ink with aid of strong acid and sodium chlorate to achieve a high yield of 80% (Yang et al., 2014). Also, Zhu and co-workers could reach a relatively high yield and introduced a magnetic hyperthermia method for rapid production of CDs on a large scale of 85 g with a yield of 60% (Zhu et al., 2020). Although these improvements in conversion yield, the procedures including separation, filtration, and drying are laborious. Yet, apparently, it is still a great challenge to produce solid CDs directly with a high conversion yield.

Herein, we demonstrate a method for the preparation of solid CDs directly with a high conversion yield. In the present synthetic process, the collagen waste was exploited as a novel carbon source not only for an economical consideration but also for the pre-existing chemical dissociation in an acid environment, since it is a critical step in the extraction of commercial collagen. The commercial collagen was produced from animal tissues (*e.g.* bovine tendon, fish skin, rat tails) by adding an excess of acetic acid and pepsin to hydrolyze the proteins into target peptides. The residual tissues can be used as a carbon source through a heat treatment to prepare CDs without extra chemical decomposition treatment. At first, the recycled collagen waste was first adjusted to a neutral pH and then dried in a freeze dryer to obtain a sponge-like precursor for calcination. The calcination temperature and time were surveyed to obtain an optimum parameter for optimal yield and quality. N and S atomic-doping CDs (N-CDs and S-CDs) were also synthesized by adding sulfuric acid or nitric acid to the collagen waste. The heteroatom doping CDs showed higher quantum yield at the same mass concertation. The prepared CDs exhibited their applications in fluorescent sensing and imaging.

## Materials and methods

### Reagents

The collagen wastes were obtained from the Laboratory of Biomimetic Nanomaterials, Department of Orthodontics, Peking University. AlCl_3_, BaCl_2_, CaCl_2_, CdCl_2_, CoCl_2_, CuCl_2_, FeCl_2_, FeCl_3_, Hg(NO_3_)_2_, KCl, NaCl, SnCl_2_, NaOH, and ethylene diamine tetraacetic acid (EDTA) were purchased from Aladdin Chemical Reagent Co., Ltd. HCl was purchased from Tianjin Kemio Chemical Reagent Co., Ltd. The reagents used in this study all were of analytical grade.

### Solid CDs fabrication

The collagen wastes were firstly washed with diluted NaOH solution (0.1 M) and distilled water until neutral, where a pH meter (PHS-3C, Yancheng Australia Tripod Instrument Manufacturing Co. Ltd., China) was used to monitor the solution acidity during the period. Then the mixture was filtered using gauze to collect the solid undergoing a freeze-drying treatment for 12 h (XYFD-1, Shanghai Xinyu Instrument Ltd., China). Then, the obtained white colored sponge-like precursor was weighed to give the accurate mass of raw material using an electronic balance (AL204, Mettler Toledo Instruments (Shanghai) Ltd., China). Subsequently, the precursor was placed in a tube furnace (GSL-1700X, Hefei Kejing Material Technology Ltd., China) to withstand calcination temperature of 350°C at a heating rate of 5°C/min under Ar atmosphere for 2 h. The obtained solid CDs could be used without further treatment. However, the purified procedures were proceeded by the following procedures to remove any insoluble aggregates in the product when the calcination temperature and time were not appropriate. First, the product was grounded and dispersed in water, while the supernatant was filtrated through a 0.22 μm ultra-filtration membrane to remove large particles. The filtrate was collected followed by a dialysis procedure using a dialysis tube (retained molecular-weight 500 Da, Shanghai Jizhi Biochemical Technology Ltd., China) in distilled water for a week to remove the residual salts. Finally, the purified liquid CDs were dried in an oven at 40°C to obtain the powder. The produced brown solids were collected and weighed to calculate the conversion yield.

### Characterization

The mass spectra were recorded on a liquid chromatograph/mass spectrometer (Thermo Fisher-Exactive Orbitrap, USA). The thermogravimetric analysis (TGA) was performed on a thermogravimetric analyzer (SDT Q600, TA Instruments, USA), with a heating rate of 5° min^−1^ in N_2_. Transmission electron microscopy (TEM) and high-resolution TEM (HR-TEM) images were recorded on a JEOL JEM 2100 transmission electron microscope (Hitachi Ltd., Tokyo, Japan) operating at an accelerating voltage of 200 kV. The instrument used to obtain X-ray powder diffraction (XRD) patterns was a Dmax-2500 X-ray diffractometer (Rigaku Ltd., Tokyo, Japan) with Cu Ka radiation (λ = 1.54 Å) at 50 kV and 200 mA at a scanning rate of 5° min^−1^. X-ray photoelectron spectroscopy (XPS) analysis was recorded on an ESCALAB MK II X-ray photoelectron spectrometer (VG Scientific Ltd., UK) using Mg as the exciting source. UV–Vis spectra were obtained on an EVOLUTION 201 Spectrophotometer (Thermo Fisher Scientific Led., Waltham, USA). Fluorescence emission spectra were recorded on a F-7000 luminescence spectrometer (Hitachi Ltd., Tokyo, Japan) at room temperature in an aqueous solution. Time-resolved fluorospectroscopy was performed using an FLS 980 spectrometer (Edinburgh Instruments Ltd., Edinburgh, UK). FT-IR spectra were performed on a Nicolet 5700 IR spectrometer (Thermo Fisher Scientific Led., Waltham, USA) in the range of 400–4000 cm^−1^. The Zeta potential measurement was performed on a Nano-ZS ZEN3600 Zetasizer (Malvern Instruments Ltd., Worcestershire, UK). The quantum yield (QY) was measured according to an established procedure (Lakowicz, 2006). Quinine sulfate in 0.1 M H_2_SO_4_ (QY = 0.54 at 360 nm) was chosen as a standard. Absolute values are calculated using the standard reference sample that had a fixed and known fluorescence QY value. To minimize reabsorption effects, absorbencies in the 10 mm fluorescence cuvette were kept under 0.1 at the excitation wavelength (360 nm).

### Detection of Fe^3+^

The detection of Fe^3+^ was performed at room temperature in PBS (10 mM, pH 3.0) buffer solution. The stock aqueous solution of twelve metal salts (AlCl_3_, BaCl_2_, CaCl_2_, CdCl_2_, CoCl_2_, CuCl_2_, FeCl_2_, FeCl_3_, Hg(NO_3_)_2_, KCl, NaCl, SnCl_2_) were prepared with concentration of 10 mM. In a typical run, a calculated amount of FeCl_3_ stock solution was added to 3 ml of CDs solution (2.5 μg/ml) with a working concentration range from 10 to 100 μM. The selectivity of metal ions was investigated based on the fluorescence quenching of CDs by adding different metal salts solutions (50 μM in the mixture) into the CDs’ suspensions, respectively.

The detection of Fe^3+^ was also performed in lake water and human blood serum. The lake water sample was taken from Tiande lake, Zhengzhou, China. Sink a clean bottle connected with a stone to 1 m depth lake, and then pull the bottle to collect the lake water sample. The human blood serum was obtained from the First Hospital Affiliated to Zhengzhou University. Different amounts of FeCl_3_ stock solutions were added in 3 ml human serum solution (volume fraction 10% in PBS), so that the concentration of Fe^3+^ in the mixture was 20, 40, 60, 80, and 100 μM, successively. The PL emission spectra were recorded after mixing for 20 min at room temperature. The error bars represent standard deviations based on the three independent measurements.

### Cell cytotoxicity assay and endocytosis

The cytotoxicity of CDs was evaluated on Bone Marrow Mesenchymal Stem Cells (BMSCs) using a modified Cell counting kit-8 (CCK-8, SAB biotech. College Park, MD, USA) assay. BMSCs were grown in α-MEM medium (Invitrogen, Carlsbad, CA, USA) containing 10% fetal bovine serum (Invitrogen) and 1% penicillin/streptomycin in humidified air with 5% CO_2_ at 37°C. To investigate cell viability, BMSCs incubated in 96-well plates were treated with control medium or medium with 1 μg/ml CDs, 10 μg/ml CDs, and 100 μg/ml CDs, and cell proliferation was assessed by a CCK-8 assay at 24 and 48 h post treatment following the manufacturer’s instruction. Optical density (OD) was recorded at 450 nm. Experiments were performed in triplicate.

CDs at a concentration of 10 μg/ml were added to the BMSCs and co-incubated for 24 h. DAPI (4′,6-diamidino-2-phenylin-dole) was used to stain the nucleus. The cells were then washed with PBS and observed using an LSM-710 confocal scanning microscope (Zeiss, Germany).

### Fluorescence imaging

Mix 0.1 g petroleum jelly with 10 µL 1 mg/ml CDs. The volunteer first washed his fingers with soap, dry them, dip a small amount of petroleum jelly mixture, and press it on a clean glass slide. The glass with fingerprint was firstly soaked in CDs ethanol/aqueous solution and stirred for 15 min, then rinsed with water and dried in air. The collagen fibers were obtained from the Laboratory of Biomimetic Nanomaterials, Department of Orthodontics, Peking University. 10 mg of collagen fibers were soaked in 5 ml of 10 μg/ml CDs and stirred for 24 h, then rinsed with water and dispersed onto a glass slide. The fluorescence pattern of the detail part of the fingerprint was observed and photographed with a Zeiss LSM-710 confocal microscope.

## Results and discussion

### Characterization of solid CDs

**SCHEME 1:**
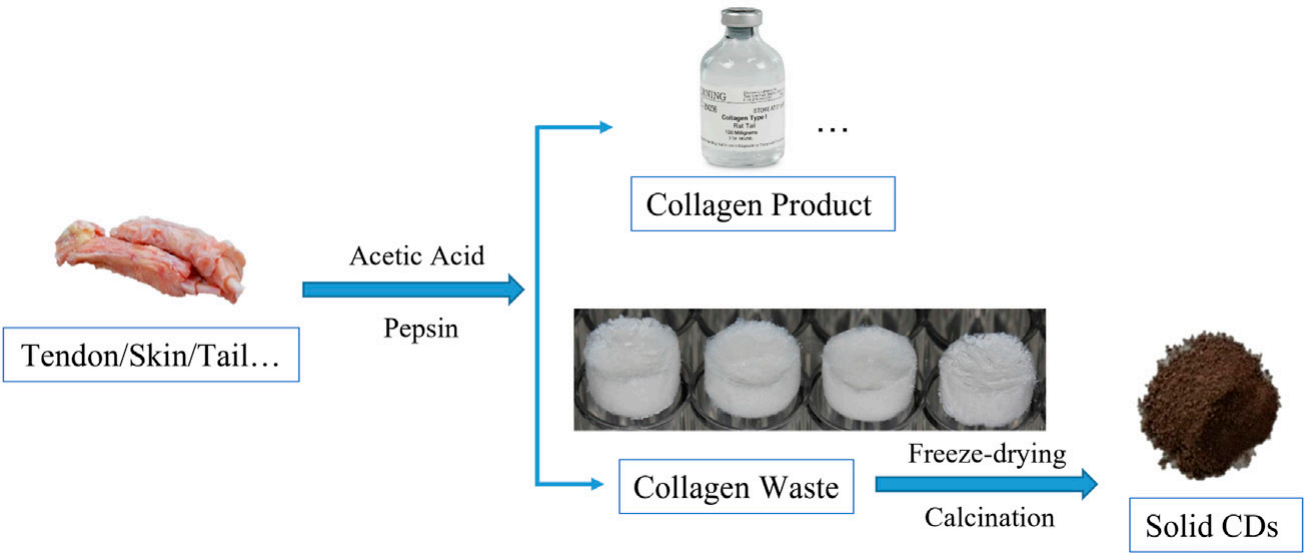
A schematic diagram of the preparation process of solid CDs. The animals tissues disaggregated into collagen product and residual tissues. The collagen waste underwent freeze-drying and calcination to convert low molecular weight peptides to solid CDs directly during the heat treatment.

The process of synthesizing CDs is schematically represented in Scheme 1. The collagen product can be produced from animals tissues rich in collagen after enzymatic hydrolysis and acidification, while the remains were collagen wastes. Solid CDs were obtained from collagen waste by freeze-drying and calcination. So far, our research group has participated in a series of studies-based collagen scaffold and their application in bone and tissue regeneration (Liu et al., 2016a; Liu, et al., 2016b; Liu et al., 2019; Wang et al., 2021; Yu et al., 2022). For example, we used molecular tropocollagen and the assembled collagen scaffold as a carbon source to prepare fluorescent CDs by hydrothermal treatment (Qiao et al., 2018). We then made full use of the residual collagen waste as a new kind of carbon source to exploit a more economical way to fabricate CDs, since the presence of acetic acid and pepsin used in the disaggregation not only provided the desired collagen tropocollagen molecules but also the favorable precursor in pyrolysis and carbonization for the synthesis of CDs. It’s worth noting that the drying process in an oven gave a much lower conversion yield of CDs, since there were lots of insoluble solids. It supposed that the freeze-drying process may play an important role in producing CDs. The sublimation of ice would create many pores in the precursor during in the freeze-drying process, while the porous structure would easily to crack and carbonize to solid CDs during the calcination procedure. Moreover, the low-pressure environment may help to reserve the functional groups to make the solid CDs soluble.

**FIGURE 1:**
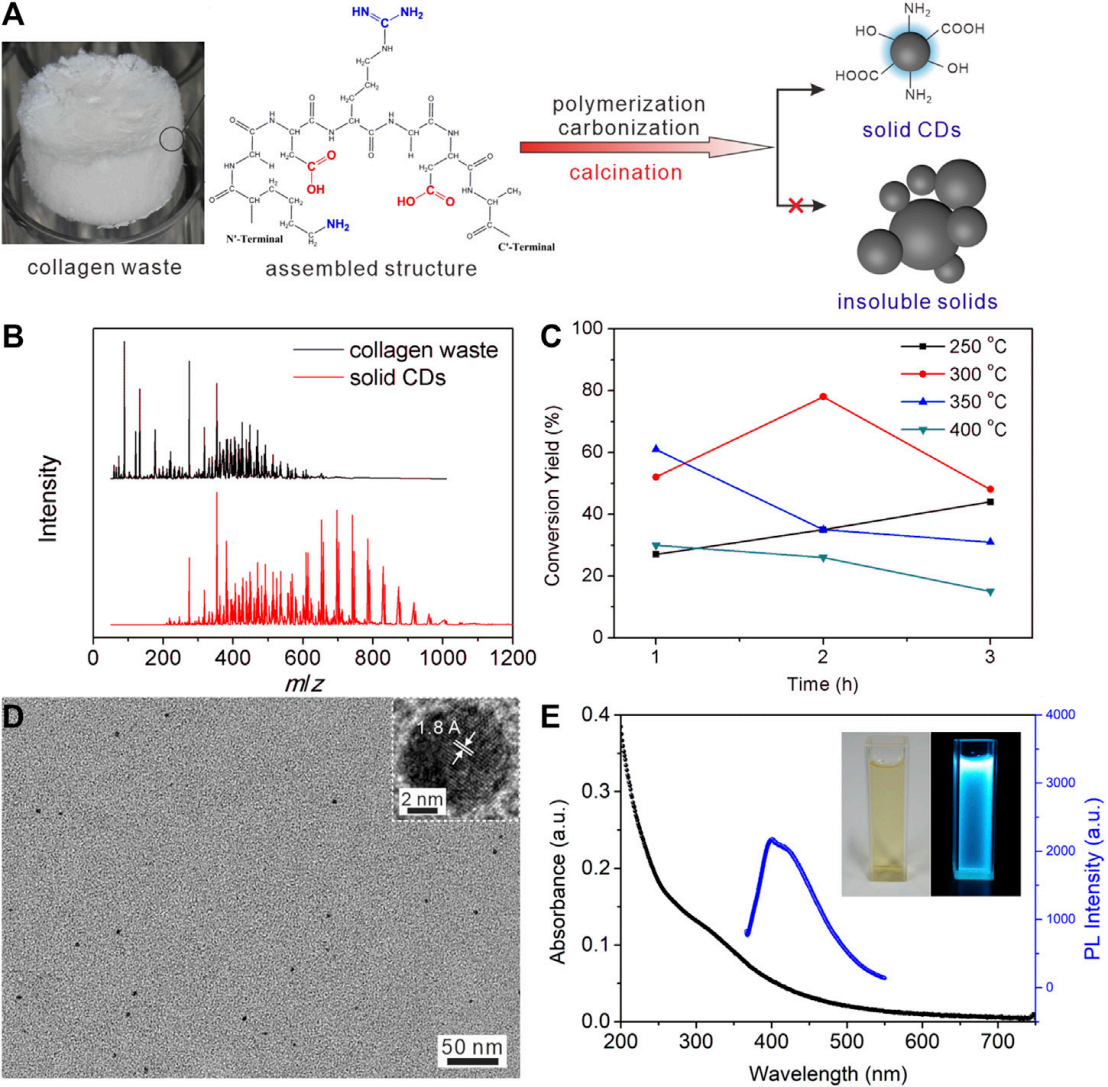
**(A)** A schematic diagram of the calcination treatment change the amino acids and peptide chains to solid CDs. **(B)** The mass spectra of collagen waste after freeze drying, and the obtained solid CDs. **(C)** The conversion yield of CDs at different calcination temperature for different time. **(D)** TEM image of CDs, the inset shows the lattice fringe of one dot. **(E)** The UV-Vis absorbance spectra and PL emission spectra of CDs aqueous solution (40 μg/ml for the former and 2.5 μg/ml for the latter).

Figure 1A schematically showed the small pieces of collagen waste tissue could convert to solid CDs directly at a high temperature supplied by the tube furnace. The mass spectra shown in Figure 1B demonstrated the presence of abundant residual amino acids and peptide chains with small molecular weight in the precursor, while the produced solid CDs were concentrated in a higher *m*/*z* zone. Supplementary Table S1 listed the main constituents in collagen waste corresponding to the peaks in the mass spectrum, from which we can see the peaks below 400 m/*z* could be assigned to alanine, L-cysteine, aspartic acid, arginine, and various fatty acids. It suggests that polymerization occurs during the heat treatment to consume the amino acids and peptide chains in the raw material. The collagen waste is an assembled structure of amino acids through intermolecular hydrogen bonds, electrostatic attractions, and hydrophobic interactions (Wegst et al., 2015). The high temperature would disaggregate the weak intermolecular interactions and lead to dehydration, polymerization, and carbonization, together with the collapsing of the assembled spongy structure of precursor. At the same time, a large amount of functional groups can be reserved on the surface to produce soluble solid CDs eventually. As our previous work (Qiao et al., 2018), the immobilized functional groups in the assembled structure, *i.e.,* carboxyl, amino, hydroxyl groups, would restrict the vibration and rotation of molecules, thereby leading to an increase of radiative transition and achieving photoluminescence property in the product.

Both calcination temperature and time had impact on the conversation yield of solid CDs during the polymerization and carbonization procedure, as shown in Figure 1C. Supplementary Figure S1 and Table 1 listed the photoluminescence (PL) intensity and yield of CDs prepared under different calcining conditions. It demonstrated that the conversion yield and PL quantum yield (QY) of CDs obtained under the condition of 300°C and 2 h calcination time were most optimal under the conditions tested. The highest conversion yield of 78% could be calculated by comparing the weight of soluble CDs product and the precursor collagen waste. The low temperature will lead to incomplete carbonization of CDs, thus reducing the conversion yield. It is concluded that the higher temperature gave rise to the growth of carbon core by carbonizing the functional groups of precursors, which is beneficial for higher production yields and QY. However, a much higher temperature will lead to increased decomposition and excessive carbonization of CDs, reducing surface amorphous portions to give insoluble agglomeration of carbon nanoparticles and reducing the yields as well. As the carbon core and surface state play a synergistic effect in contributing to the PL property of CDs, the maximum conversion yield and PL QY were approached at the in-between temperature range of 250–400°C where both the graphite core and amorphous shell structures coexist. This is similar to previous reports (Krysmann et al., 2012; Jones et al., 2017; Wu et al., 2018). The TGA analysis of solid CDs shown in Supplementary Figure S2 exhibited a H_2_O evaporation weight loss below 100°C and another weight loss between 280 and 390°C, which is presumably due to the loss of CO and CO_2_ from the oxygen-containing functional groups. It is concluded that the intermolecular interactions and functional groups have been decomposed stepwise during the calcination procedure. The appropriate calcination temperature would maintain plenty of functional groups as well as crystalline core to produce soluble CDs. The calcination time had a comparable impact on the formation of CDs. The longer reaction time would result in a higher carbonization degree, which promotes the growth of the carbon core and thus enhances the fluorescence properties of the CDs. On a lower calcination temperature 250°C, the longer calcination time certainly generated higher yields. On 300°C, both the conversion yield and QY of CDs exhibited a trend of first increasing and then decreasing with the increase of reaction time. In contrast, however, above 300°C the yields significantly decreased along with the reaction time. Supplementary Figure S3 showed the optical photograph of the product collected from the cooled-down tube furnace under the optimal calcination condition. The solid CDs appeared to be a brown-colored powder with single production of 5.87 g. Furthermore, they can dissolve in water completely to make a stock solution with mass concertation of 1 mg/ml. Table 2 listed the comparison of routes for producing CDs on large scale (Park et al., 2014; Yang et al., 2014; Jones et al., 2017; Ding et al., 2018; Zhu et al., 2020; Niu et al., 2021; Ding et al., 2022), suggesting our method might have an economical consideration with high conversion yield.

**TABLE 1:** The PL intensity and conversion yield of CDs prepared under different calcining conditions.

Calcination conditions (°C)		Conversion yield (%)	PL QY (%)
250	1 h	27	2.5
	2 h	35	5.1
	3 h	44	3.4
300	1 h	52	2.5
	2 h	78	7.3
	3 h	48	4.4
350	1 h	61	2.8
	2 h	35	5.5
	3 h	31	5.0
400	1 h	30	6.1
	2 h	26	2.8
	3 h	15	0.5

**TABLE 2:** Methods for the large-scale production of CDs.

	Raw materials	Methods	State	Reaction conditions	Production yields	Conversion yields (%)	Ref.
Chemicals	Chinese ink, HNO_3_, H_2_SO_4_, NaClO_3_	Chemical oxidation	Liquid	15°C, 5 h	120 g	80	Yang et al. (2014)
	Citrate, carbamide, Fe_3_O_4_	Magnetic hyperthermia	Liquid	450 kHz, 1 h	85 g	60	Zhu et al. (2020)
	Phloroglucinol, boric acid	Solid heating	Solid	200°C, 3 h	-	75	Niu et al. (2021)
	*o*-phenylenediamine, KCl	Solid heating	Solid	180°C, 6 h	1.9 g	72	Ding et al. (2022)
Biomass	Food waste	Ultrasound	Liquid	40 kHz, 40 min	120 g	0.12	Park et al. (2014)
	Chia seeds	Calcination	Solid	350°C, 6 h	-	10	Jones et al. (2017)
	Alkali lignin, HNO_3_	Hydrothermal	Liquid	180°C, 12 h	0.63 g	21	Ding et al. (2018)
	Collagen waste	Calcination	Solid	300°C, 2 h	5.87 g	78	This work

Figure 1D exhibits a morphological characterization of CDs solution, exhibiting a quasi-spherical shape with diameter of 4.1 ± 2.2 nm. The HR-TEM shown in the inset exhibited a clear lattice fringe of 1.8 Å that can be corresponded to the (102) plane of graphite. The XRD pattern shown in Supplementary Figure S4 exhibited a quite broad peak around 24° corresponding to the predominantly interlayer-stacking reflection of aromatic structures (Li et al., 2020), while the adsorbed surface functional groups should be responsible for the poor crystallization of the solid CDs. The crystallization morphology and structure further declared the amino acids and peptide chains turn to carbonization directly under high temperature in the reactor to generate CDs. The calcination temperature and time were surveyed to demonstrate the optimal experimental conditions for a higher conversion yield of CDs. The photos of CDs solution at concentration of 0.1 mg/ml under visible and UV light (365 nm) declared that the pale-yellow solution gave a bright blue fluorescence under UV irradiation (the inset in Figure 1E). The QY of thus formed CDs was calculated as 7.3%. The absorption profile of CDs dispersion shown in Figure 1E exhibited strong absorption in the UV region and a shoulder peak centered around 330 nm, which can be assigned to *n*-π* transitions of C=O and *π*-π* transitions of C=C in the CDs surface functional groups. The PL spectrum recorded when excited with 340 nm showed a broad emission around 413 nm, which was consistent with the observed blue fluorescence. The synthesized CDs also exhibited a strong dependence of the emission on the excitation wavelength (Supplementary Figure S5), which corresponds with a previous report (Anuar et al., 2021). The inhomogeneous particle size and the surface state of CDs can be responsible for the excitation-dependent PL property of CDs (Jiang et al., 2012; Yan et al., 2020).

**FIGURE 2:**
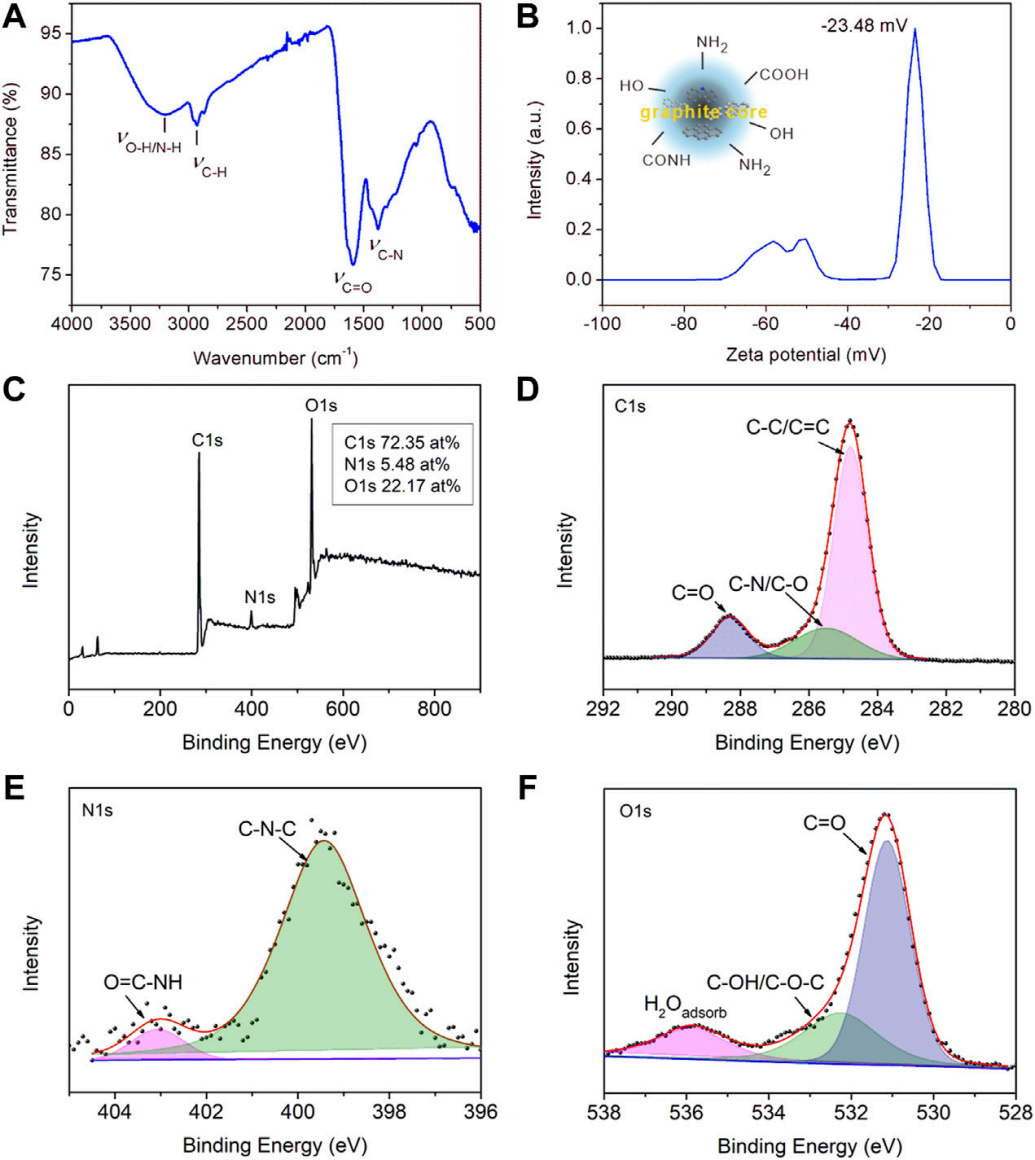
**(A)** FT-IR spectra of solid CDs. **(B)** The zeta potential of CDs aqueous solution. **(C)** The survey XPS spectra of CDs. **(D)** High resolution spectra of C1s, **(E)** N1s, and **(F)** O1s.

The detailed surface functional groups were surveyed by FT-IR spectra, zeta potential, and XPS spectra, as shown in Figure 2. As shown in Figure 2A, the most intense band at 1654 cm^−1^ represented the stretching vibration of C=O groups. The broad bands at about 3,000–3,500 cm^−1^ were due to stretching vibrations of–OH or–NH, which originated from the terminal hydroxyl or amino groups in the CDs. The peaks around 2927 cm^−1^ and 2827 cm^−1^ could be assigned to asymmetry and symmetry stretching vibrations of C–H groups, while the stretching vibration of C-N gave the absorption peak at 1377 cm^−1^. The FT-IR spectra suggested that there were abundant amino, carboxyl, and other hydrophilic groups on its surface, contributing to good water solubility. Zeta potential referred to the potential of shear plane and is an important indicator of stability of colloid dispersion system. The surface properties and electron density of CDs were studied as shown in Figure 2B. The Zeta potential value was −23.48 mV, indicating the electron-rich nature of the surface of CDs, originating from the surface nitrogen, and oxygen-containing groups. The XPS results worked in concert with the above FT-IR and Zeta potential, as shown in Figures 2C–F. CDs were mainly composed of C, N, and O elements, among which the atomic percentages are 72.35 at%, 5.48 at%, and 22.17 at%, respectively. The peak compositions of the three elements were further analyzed by high resolution XPS spectra. The deconvolution analysis of C1s spectrum shows three peaks at 284.7, 285.4, and 288.3 eV, belonging to C-C/C=C, C-N/C-O, and C=O groups, respectively. The N1s spectrum had two peaks at 399.3 and 400.2 eV, corresponding to the C-N-C and O=C-NH groups, respectively (Lv et al., 2020). The two peaks in O1s spectrum located at 531.1 and 535.5 eV can be assigned to C=O and C-OH/C-O-C groups. These results further indicated that the prepared CDs contained many hydrophilic groups such as hydroxyl and carboxyl groups, not only contributing to their water solubility but also providing lone pair electrons for the coordination of metal ions, bringing avenues for their sensing applications.

Fluorescence lifetime and recombination channels were used to reveal the PL mechanism. The time-resolved fluorescence decay spectra of CDs were collected as shown in Supplementary Figure S6. The attenuation curve can be fitted by a three exponential model, *R(t)* = *B*_*1*_ *e* ^*(-t/τ1)*^+*B*_*2*_ *e* ^*(-t/τ2)*^+*B*_*3*_ *e* ^*(-t/τ3)*^. The time parameters and corresponding weights in the fitting model are listed in Table 3, indicating the mean lifetime (*τ*_mean_) is 5.43 ns. There is a fast component (∼1 ns) and two slow components (∼4 and ∼11 ns), which are attributed to the inherent state emission and the defective state emission, respectively. The prepared CDs had more weight on the slow component, suggesting the PL mechanism may defect state luminescence.

**TABLE 3:** Time constants (*τ*_1_, *τ*_2_, *τ*_3_) and the fractional weights of the various decay time components (*α*_1_, *α*_2_, *α*_3_) of CDs. The average lifetime *τ*_mean_ was calculated using the following equation: $\tau_{\text{mean}}=\frac{\sum^{\text{​}}\alpha_{\text{i}}\tau_{\text{i}}^{2}}{\sum^{\text{​}}{\text{α}}_{\text{i}}{\text{τ}}_{\text{i}}}$ .

Sample	*α*_1_	*τ*_1_	*α*_2_	*τ*_2_	*α*_3_	*τ*_3_	*τ*_mean_
CDs	20.62%	1.51ns	54.55%	4.26ns	24.83%	11.25ns	5.43ns

Stability is an important aspect to evaluate one fluorescent material, since the changing external environment (such as solution pH, ionic strength, and UV irradiation time) may affect the luminescence characteristics. Therefore, the influences of these environmental factors were analyzed as shown in Supplementary Figure S7. In the pH range of 2–5, the PL intensity presented a tenuous increase with the decrease of solution acidity, while fluctuates slightly during the pH range of 7–11. It is suggested that the variation of PL intensity with the solution pH may be caused by the electron transitions of π-π* and n-π* in the graphene nanodomains of CDs by refilling or depleting the valence bands. This result showed that CDs can survive a good fluorescence property during a wide pH window. The influence of ionic strength on the PL intensity was studied by changing NaCl concentration in CDs dispersion. The PL intensity of CDs decreases slightly, however, still maintains a high value, when NaCl concentration increased from 0 to 1.0 M. Furthermore, the PL emission intensity of CDs gradually weakens and tends to be stable with the UV radiation time. The fluorescent brightness was slightly affected by the UV illumination, indicating CDs had strong photobleaching resistance that can be stood a long time of test and modification. These results illustrate the prepared CDs had good stability under the harsh environment, which makes them appropriate in the actual physiological environment.

### N-CDs and S-CDs

**FIGURE 3:**
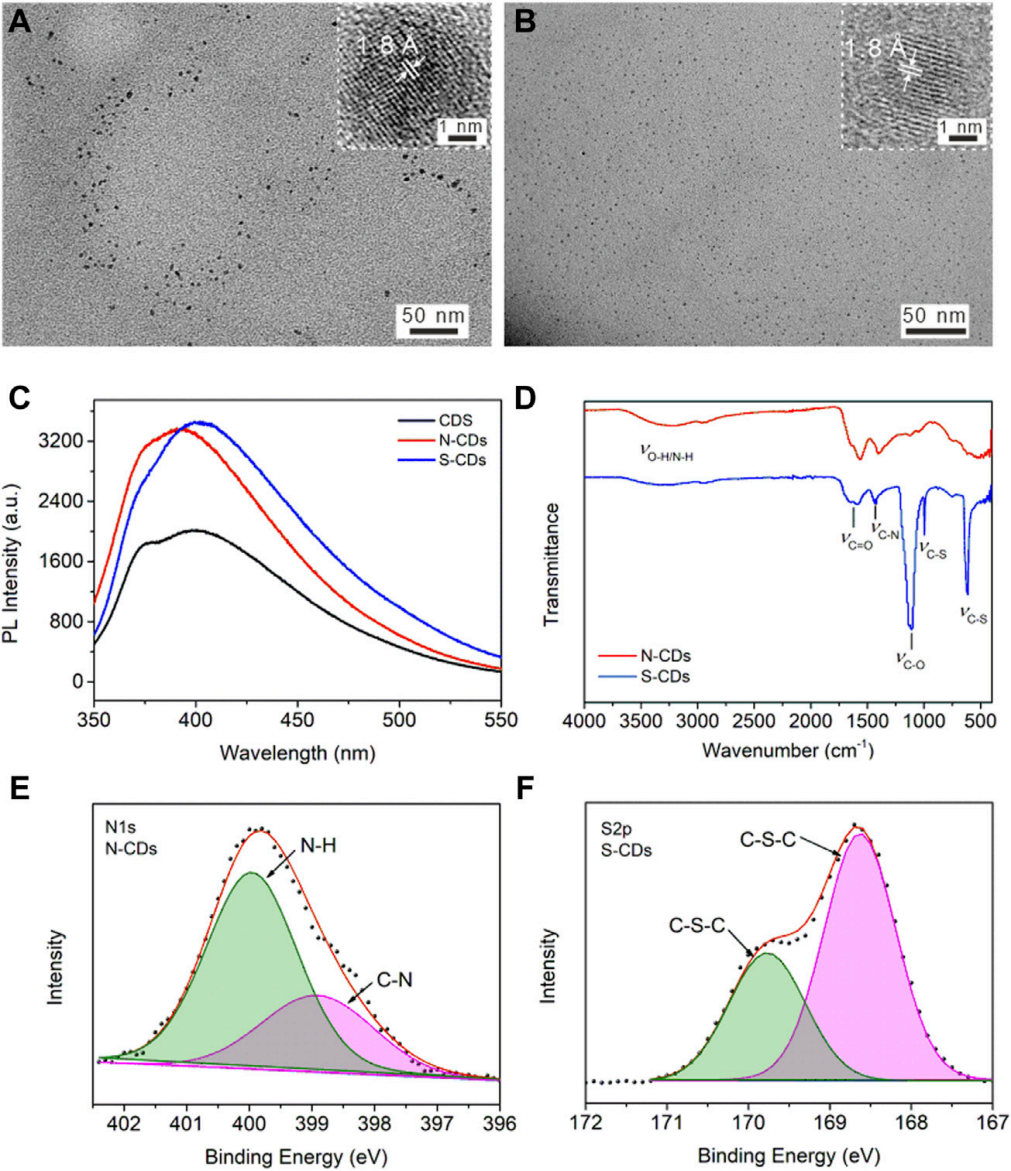
TEM image of **(A)** N-CDs, and **(B)** S-CDs. The inset shows the lattice fringe of one dot. **(C)** PL emission spectra of CDs, N-CDs, and S-CDs aqueous solution with same concertation of 2.5 μg/ml **(D)** FT-IR spectra of N-CDs and S-CDs. High resolution spectra of **(E)** N1s for N-CDs, and **(F)** S2p for S-CDs.

Heteroatomic doping is an efficient and convenient method for adjusting optical properties and surface passivation of CDs. N atom is often doped into the carbon material since it has a similar atomic size to carbon atom and is more electronegative than carbon atom, which can enhance the PL of CDs by creating new defects on their surface (Guo et al., 2018; Zhu et al., 2019). It is reported that the doping S atoms in the CDs structure can provide an energy trap state, in which photoexcited electrons can be captured thus avoiding self-quenching (Xu et al., 2015; Wang et al., 2019). Our method can be generalized to produce N-CDs and S-CDs by immersing the collagen waste in nitric acid or sulfuric acid in advance. The TEM images of prepared N-CDs and S-CDs were shown in Figures 3A,B. The size distribution of N-CDs and S-CDs were 4.2 ± 1.7 nm and 2.2 ± 1.2 nm, respectively. The HR-TEM images exhibited a clear lattice fringe of 1.8 Å, which was similar to that in CDs (the inset in Figure 1D). The PL intensity of doping CDs exhibited enhanced intensity compared with the undoped CDs, as shown in Figure 3C. The PL intensity of N-CDs and S-CDs was 1.62 times and 1.26 times higher than that of undoped CDs, respectively. The calculated QY also improved to 12.6% for N-CDs and 10.4% for S-CDs, respectively. The results indicated that the PL properties of doped CDs were significantly improved. The FT-IR spectra shown in Figure 3D illustrated the abundant function groups in N-CDs and S-CDs. The XPS result (Supplementary Figure S8) illustrated that the N content has improved to 13.58% from 5.48% in the N-CDs compared with that of original undoped ones. Figure 3E showed the deconvolution N_1s_ spectrum for N-CDs that had two peaks at 398.8 and 400.1 eV, corresponding to pyridine N and N-H groups, respectively. As for S-CDs, they were mainly composed of C, N, O, and S elements, among which the atomic percentages were 28.95%, 5.53%, 53.36, and 12.16%, respectively. The S_2p_ spectrum shown in Figure 3F can be split into two peaks at 168.6 and 169.8 eV, which were characteristic peaks of S_2p3/2_ and S_2P1/2_, indicating the presence of the C-S-C group. Compared with the element content in the undoped CDs, N and S elements were successfully doped in the nanocarbon materials through acidizing with nitric acid and sulfuric acid, indicating they provided sufficient N and S sources during the process of acidizing carbon precursor.

### Sensing of Fe^3+^

**FIGURE 4:**
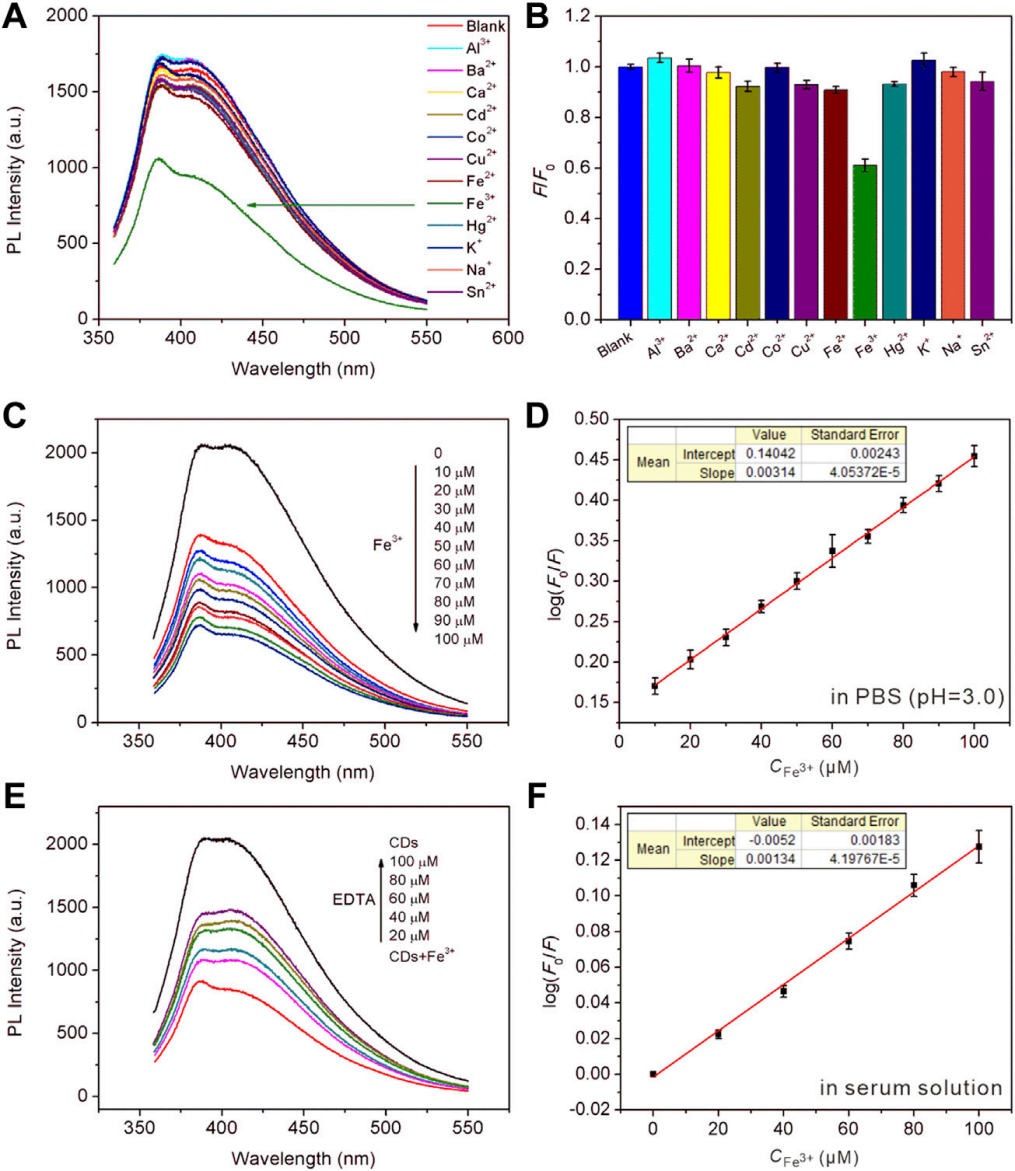
**(A,B)** The selectivity survey of CDs towards different metal ions, [M^n+^] = 50 μM. **(C)** PL intensity attenuation of CDs in the presence of increasing concentration of Fe^3+^. **(D)** A linear calibration plot of log(*F*_0_/*F*) versus the concentration of Fe^3+^ over the range of 10–100 μM. The inset shows the fitting equation. **(E)** The fluorescence recovery plot by adding increasing amount of EDTA. **(F)** A linear calibration plot of the concentration of Fe^3+^ in the serum medium. The inset shows the fitting equation.

Iron ion is one of the most important and abundant transition metal ions in biological systems, playing an indispensable role in many physiological and pathological processes, such as cell metabolism, enzyme catalysis, and DNA and RNA synthesis. Moreover, the iron ion is often used in agricultural and industrial processes, and it is also involved in pollution of crops and the environment. Therefore, it is of great significance to develop a simple method for the efficient detection of iron ions. The prepared CDs can serve as a fluorescence sensing platform for Fe^3+^ in acid medium with high sensitivity and specificity. The undoped CDs were selected in the fluorescent sensing from the point of view of generalizability and ease of preparation. Figure 4A recorded the PL intensity of CDs towards adding different metal ions (Al^3+^, Ba^2+^, Ca^2+^, Cd^2+^, Co^2+^, Cu^2+^, Fe^2+^, Fe^3+^, Hg^2+^, K^+^, Na^+^, Sn^2+^). As can be seen from Figure 4B, the PL intensity of CDs mixed with Fe^3+^ decreased significantly under acidic conditions (pH = 3), while other metal ions did not impact significantly. Supplementary Figure S9 indicates that the PL intensity hardly changed in the medium of mixed metal ions without Fe^3+^, while decreased sharply only when Fe^3+^ in the presence of mixed solution. It was reported that the interference of other metal ions on the PL intensity of CDs can be shielded under acidic conditions (Zhu et al., 2017). The *K*_sp_ of Fe^3+^ complexing with the hydroxyl groups on the surface of CDs was far less than that of other metal ions under acidic conditions. Fe^3+^ can preferentially bind with CDs to produce specific fluorescence quenching, resulting from photoinduced excitation energy transfers to the empty *d*-orbital of Fe^3+^ and releases in a non-radiative way. As shown in Figure 4C, the PL intensity decreased with the addition of increasing concentration of Fe^3+^. This phenomenon occurs because more hydroxyl groups coordinated with the metal ions, which further changed the surface defects of CDs leading to significantly weaken and quenching of PL intensity with the increase of Fe^3+^ concentration. Figure 4D showed the linear response curve of relative reduced PL intensity between CDs dispersion with increasing Fe^3+^ concentrations. The results showed a linear relationship between log(*F*_0_/*F*) and Fe^3+^ concentration in the range of 10–100 μM, which can be written as log(*F*_0_/*F*) = 0.14042 + 0.00314[Fe^3+^] (*R* ^2^ = 0.998), and the detection limit is 0.04 μM. Supplementary Figure S10 showed the Zeta potential of CDs before and after addition of Fe^3+^, it changed from −23.48 to 23.55 mV. This is because Fe^3+^ can coordinate with the functional groups on the surface of CDs, resulting in the decrease of lone-pair electrons and conversion to positive potential. Ethylene diamine tetraacetic acid (EDTA) solution was added to the above CDs-Fe^3+^ system, as can be seen from Figure 4E, the PL intensity of CDs gradually recovered with the increasing amount of EDTA. Since EDTA is a strong chelating reagent towards metal ions that can capture Fe^3+^ from the CDs surface, which makes the PL intensity restore. Supplementary Figure S11 and Figure 4F showed the PL intensity attenuation curves and relationship between CDs PL intensity and Fe^3+^ concentrations in the human serum solution system (human serum volume fraction 10%), respectively. There is a linear relationship with the gradual increase of Fe^3+^ concentration as well, log(*F*_0_/*F*) = -0.0052 + 0.00134[Fe^3+^] (*R* ^2^ = 0.996), suggesting the florescent CDs are suitable for sensing applications in the complex physiological environment. We also conducted recovery tests to assess the accuracy of CDs sensing platform in real lake water samples. The content and recovery results were shown in Table 4. The recovery rate of the fluorescence detection system was 96.47–101.8%, and the relative standard deviation was less than 3.6% (*n* = 3), proving that the fluorescence sensing platform based on CDs is an accurate and reliable method for the detection of Fe^3+^ in environmental samples.

**TABLE 4:** Determination of Fe^3+^ in lake water samples (*n* = 3).

Fe^3+^	Added (μM)	Found (μM)	Recovery (%)	RSD (%)
Lake water	5.00	5.08	101.60	1.59
	15.00	14.47	96.47	3.59
	25.00	25.45	101.80	1.78

RSD, relative standard deviation.

### Cytotoxicity and fluorescence imaging

**FIGURE 5:**
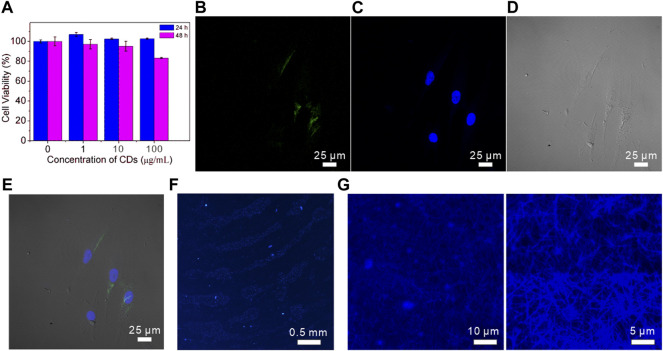
**(A)** CCK-8 assay results for the viability of MSCs cells in different concentrations of CDs; **(B–E)** CLSM images of MSCs cells co-incubated with CDs for 24 h. Images were captured with **(B)** *λ*_ex_ = 488 nm, **(C)** *λ*_ex_ = 430 nm laser; **(D)** bright filed, **(E)** merged image; **(F)** fluorescent images of CDs-stained fingerprints on a glass sheet; **(G)** fluorescent images of CDs-stained collagen fibrils at low and high magnification.

As a fluorescence nanomaterial, CDs have a promising application in bioimaging and biomedicine. The cytotoxicity of prepared CDs is an important measurable indicator to investigate the biological safety and biocompatibility. The CCK-8 assays were conducted to evaluate the cell viability by co-incubation of CDs and BMSCs for 24 and 48 h (Figure 5A). It was found that the cell proliferation rate mildly increased when the CDs existed in matrix compared with the control condition. Further incubation indicated that the CDs could slightly suppress the cell growth after 48 h. Above 90% of cells still survived with 10 μg/ml CDs for 48 h. When the CDs concentration reached 100 μg/ml, the viability of BMSCs reduced to 83% compared with PBS. Similar to other reports (Jiao et al., 2020; Yue et al., 2021; Wang et al., 2022), it suggested that the CDs have mild cytotoxicity after cells exposed to a higher concentration of CDs for 48 h.

In view of the good biocompatibility of 10 μg/ml CDs within 24 h co-incubation, we investigated their cellular uptake activity using a confocal scanning microscope. As shown in Figure 5B, BMSCs treated with the CD solution showed green fluorescence when stimulated with a 488 nm laser. The cell nucleus was stained with DAPI to exhibit bright blue fluorescence under 430 nm excitation (Figure 5C). The cell morphology was clearly recognized in the bright field image shown in Figure 5D. The merged image shown in Figure 5E suggested that the CDs had entered the BMSCs and were reserved in the cytoplasm.

To further explore the fluorescence imaging application of CDs, the fluorescence image of fingerprints labeled with CDs was collected under excitation of 365 nm laser. Figure 5F clearly showed the morphology of papillary ridges in the fingerprint, including branching and crossing, which are effective information to distinguish and identify individuals. Figure 5G exhibited the fluorescence images of collagen fibrils that were stained by CDs to determine the hydrophilicity of collagen fibrils with a diameter of about 200–500 nm. The collagen fibrils are assembled tropocollagen molecules, which have wild applications in bone or tissue regeneration and repair (Liu et al., 2016b). In addition, CDs can also be used as a biological imaging agent to meet the requirements of biological system visualization experiments due to their low toxicity and biocompatibility.

## Conclusion

In the present work, the solid CDs were firstly fabricated by carbonization of collagen waste under 300°C for 2 h without extra chemical or physical treatments. The originally added acetic acid and pepsin for previous extraction procedures helped disaggregate macromolecular protein into assembled peptide chains and amino acids, thus giving preferential precursor for polymerization and carbonization of nanocarbon cores under high temperature. The method can also be promoted in preparing N and S atoms doping CDs using nitric acid or sulfuric acid as additional acidifiers. The fluorescence sensor platform is constructed by exploiting the prepared CDs in the application of highly sensitive detection of Fe^3+^. The cytotoxicity assays were conducted to evaluate the low toxicity and biocompatibility of the prepared CDs. The further fluorescence imaging of fingerprints and collagen fibrils verified the imaging application of CDs. It is a valuable work since the production of solid CDs directly can save steps in transferring huge volumes of the liquid product if carry a large-scale synthesis. The reutilization of collagen waste which should be otherwise discarded opens up exciting carbon sources for developing florescent nanocarbon materials and applications.

## Data Availability

The original contributions presented in the study are included in the article/Supplementary Material, further inquiries can be directed to the corresponding authors.

## References

[B1] Anuar N. K. K. Tan H. L. Lim Y. P. Sóaib M. S. Bakar N. F. (2021). A Review on Multifunctional carbon-dots synthesized from biomass waste: Design/fabrication, characterization and applications. Front. Energy Res. 9, 626549. 10.3389/fenrg.2021.626549

[B2] Ding H. Zhou X. Zhang Z. Zhao Y. Wei J. S. Xiong H. M. (2022). Large scale synthesis of full-Color emissive carbon dots from A single carbon source by A Solvent-Free method. Nano Res. 15 (4), 3548–3555. 10.1007/s12274-021-3891-0

[B3] Ding Z. Li F. Wang X. Sun R. (2018). Gram-scale synthesis of single-crystalline graphene quantum dots Derived from Lignin biomass. Green Chem. 20 (6), 1383–1390. 10.1039/C7GC03218H

[B4] Guo X. Xu L. Zhang L. Wang H. Wang X. Liu X. (2018). One-pot solid Phase pyrolysis synthesis of highly fluorescent nitrogen-doped carbon dots and the interaction with human serum Albumin. J. Lumin. 196, 100–110. 10.1016/j.jlumin.2017.12.029

[B5] Jiang J. Li S. Cui H. (2012). Amino acids as the source for producing carbon Nanodots: Microwave assisted one-step synthesis, Intrinsic photoluminescence property and intense Chemiluminescence Enhancement. Chem. Commun. 48 (77), 9634–9636. 10.1039/C2CC34612E 22908119

[B6] Jiao Y. Guo Y. Fan Y. Wang R. Li X. Wu H. (2020). Triggering of Apoptosis in Osteosarcoma 143B cell line by carbon quantum dots via the Mitochondrial Apoptotic signal Pathway. Biomed. Res. Int. 2020, 1–12. 10.1155/2020/2846297 PMC7369657 32733936

[B7] Jones S. S. Sahatiya P. Badhulika S. (2017). One step, high yield synthesis of Amphiphilic carbon quantum dots Derived from chia seeds: A Solvatochromic study. New J. Chem. 41 (21), 13130–13139. 10.1039/C7NJ03513F

[B8] Krysmann M. J. Kelarakis A. Dallas P. Giannelis E. P. (2012). Formation mechanism of Carbogenic nanoparticles with dual photoluminescence emission. J. Am. Chem. Soc. 134 (2), 747–750. 10.1021/ja204661r 22201260

[B9] J. R. Lakowicz (Editor) (2006). Principles of fluorescence spectroscopy. 3rd ed. (New York: Publishers: Springer). 10.1007/978-0-387-46312-4

[B10] Li G. Wang B. Zhang J. Wang R. Liu H. (2020). Er-doped g-C_3_N_4_ for Photodegradation of Tetracycline and Tylosin: High Photocatalytic activity and low Leaching toxicity. Chem. Eng. J. 391, 123500. 10.1016/j.cej.2019.123500

[B11] Liu Y. Liu S. Luo D. Xue Z. Yang X. Gu L. (2016a). Hierarchically Staggered Nanostructure of Mineralized collagen as a bone-Grafting scaffold. Adv. Mat. 28 (39), 8740–8748. 10.1002/adma.201602628 27530607

[B12] Liu Y. Luo D. Wang T. (2016b). Hierarchical structures of bone and Bioinspired bone tissue Engineering. Small 12 (34), 4611–4632. 10.1002/smll.201600626 27322951

[B13] Liu Y. Luo D. Yu M. Wang Y. Jin S. Li Z. (2019). Thermodynamically Controlled self-Assembly of Hierarchically Staggered architecture as an Osteoinductive Alternative to bone Autografts. Adv. Funct. Mat. 29 (10), 1806445. 10.1002/adfm.201806445

[B14] Lu W. Qin X. Liu S. Chang G. Zhang Y. Luo Y. (2012). Economical, green synthesis of fluorescent carbon nanoparticles and their Use as Probes for sensitive and selective detection of Mercury(II) ions. Anal. Chem. 84 (12), 5351–5357. 10.1021/ac3007939 22681704

[B15] Lv X. Man H. Dong L. Huang J. Wang X. (2020). Preparation of highly crystalline nitrogen-doped carbon dots and their application in Sequential fluorescent detection of Fe^3+^ and Ascorbic acid. Food Chem. x. 326, 126935. 10.1016/j.foodchem.2020.126935 32447160

[B16] Niu X. Song T. Xiong H. (2021). Large scale synthesis of red emissive carbon dots powder by solid state reaction for fingerprint identification. Chin. Chem. Lett. 32 (6), 1953–1956. 10.1016/j.cclet.2021.01.006

[B17] Park S. Y. Lee H. U. Park E. S. Lee S. C. Lee J.-W. Jeong S. W. (2014). Photoluminescent green carbon Nanodots from food-waste-Derived sources: Large-scale synthesis, properties, and Biomedical applications. ACS Appl. Mat. Interfaces 6 (5), 3365–3370. 10.1021/am500159p 24512145

[B18] Qiao X. Luo D. Yu M. Zhang T. Cao X. Zhou Y. (2018). A Precisely assembled carbon source to synthesize fluorescent carbon quantum dots for sensing Probes and bioimaging agents. Chem. Eur. J. 24 (9), 2257–2263. 10.1002/chem.201705310 29231271

[B19] Qin X. Lu W. Asiri A. M. Al-Youbi A. O. Sun X. (2013a). Green, low-cost synthesis of photoluminescent carbon dots by hydrothermal treatment of willow bark and their application as an effective Photocatalyst for fabricating Au nanoparticles–reduced graphene Oxide Nanocomposites for glucose detection. Catal. Sci. Technol. 3 (4), 1027–1035. 10.1039/C2CY20635H

[B20] Qin X. Lu W. Asiri A. M. Al-Youbi A. O. Sun X. (2013b). Microwave-assisted rapid green synthesis of photoluminescent carbon Nanodots from flour and their applications for sensitive and selective detection of Mercury(II) ions. Sensors Actuators B Chem. 184, 156–162. 10.1016/j.snb.2013.04.079j.snb.2013.04.079

[B21] Wang C. Wang Y. Shi H. Yan Y. Liu E. Hu X. (2019). A strong blue fluorescent nanoprobe for highly sensitive and selective detection of Mercury(II) based on Sulfur doped carbon quantum dots. Mat. Chem. Phys. 232, 145–151. 10.1016/j.matchemphys.2019.04.071

[B22] Wang M. Liu M. Nong S. Song W. Zhang X. Shen S. (2022). Highly luminescent Nucleoside-based N, P-doped carbon dots for sensitive detection of ions and bioimaging. Front. Chem. 10, 906806. 10.3389/fchem.2022.906806 35747344 PMC9210210

[B23] Wang Y. Jin S. Luo D. He D. Shi C. Zhu L. (2021). Functional regeneration and repair of tendons using Biomimetic scaffolds Loaded with recombinant Periostin. Nat. Commun. 12, 1293. 10.1038/s41467-021-21545-1 33637721 PMC7910464

[B24] Wareing T. C. Gentile P. Phan A. N. (2021). Biomass-based carbon dots: Current Development and Future Perspectives. ACS Nano 15 (10), 15471–15501. 10.1021/acsnano.1c03886 34559522

[B25] Wegst U. G. K. Bai H. Saiz E. Tomsia A. P. Ritchie R. O. (2015). Bioinspired structural materials. Nat. Mat. 14, 23–36. 10.1038/nmat4089 25344782

[B26] Wu S. Li W. Zhou W. Zhan Y. Hu C. Zhuang J. (2018). Large-scale one-step synthesis of carbon dots from Yeast Extract powder and construction of carbon dots/PVA fluorescent shape memory material. Adv. Opt. Mat. 6 (7), 1701150. 10.1002/adom.201701150

[B27] Xu Q. Pu P. Zhao J. Dong C. Gao C. Chen Y. (2015). Preparation of highly photoluminescent Sulfur-doped carbon dots for Fe(III) detection. J. Mat. Chem. A 3 (2), 542–546. 10.1039/C4TA05483K

[B28] Xu X. Ray R. Gu Y. Ploehn H. J. Gearheart L. Raker K. (2004). Electrophoretic analysis and purification of fluorescent single-Walled carbon nanotube Fragments. J. Am. Chem. Soc. 126 (40), 12736–12737. 10.1021/ja040082h 15469243

[B29] Yan H. Ni H. Yang Y. Shan C. Yang X. Li X. (2020). Smart nanoprobe based on two-photon sensitized terbium-carbon dots for dual-mode fluorescence thermometer and antibacterial. Chin. Chem. Lett. 31 (7), 1792–1796. 10.1016/j.cclet.2019.12.022

[B30] Yang S. Sun J. Li X. Zhou W. Wang Z. He P. (2014). Large-scale fabrication of Heavy doped carbon quantum dots with Tunable-photoluminescence and sensitive fluorescence detection. J. Mat. Chem. A 2 (23), 8660–8667. 10.1039/C4TA00860J

[B31] Yu M. Luo D. Qiao J. Guo J. He D. Jin S. (2022). A hierarchical bilayer architecture for complex tissue regeneration. Bioact. Mat. 10, 93–106. 10.1016/j.bioactmat.2021.08.024 PMC8636921 34901532

[B32] Yue J. Yu L. Li L. Liu P. Mei Q. Dong W. F. (2021). One-step synthesis of green fluorescent carbon dots for Chloride detecting and for bioimaging. Front. Chem. 9, 718856. 10.3389/fchem.2021.718856 34604169 PMC8484530

[B33] Zhu R. Huang W. Ma X. Zhang Y. Yue C. Fang W. (2019). Nitrogen-doped carbon dots-V_2_O_5_ Nanobelts sensing platform for sensitive detection of Ascorbic acid and Alkaline Phosphatase activity. Anal. Chim. Acta X. 1089, 131–143. 10.1016/j.aca.2019.08.061 31627810

[B34] Zhu X. Zhang Z. Xue Z. Huang C. Shan Y. Liu C. (2017). Understanding the selective detection of Fe^3+^ based on graphene quantum dots as fluorescent Probes: The *K* _sp_ of a metal Hydroxide-assisted mechanism. Anal. Chem. 89 (22), 12054–12058. 10.1021/acs.analchem.7b02499.analchem.7b02499 29050471

[B35] Zhu Z. Cheng R. Ling L. Li Q. Chen S. (2020). Rapid and large-scale production of Multi-fluorescence carbon dots by a magnetic hyperthermia method. Angew. Chem. Int. Ed. 59 (8), 3099–3105. 10.1002/anie.201914331 31828854

