# Supplementary material for: Direct preparation of solid carbon dots by pyrolysis of collagen waste and their applications in fluorescent sensing and imaging
Source: Front Chem. 2022 Sep 12;10:1006389. doi: 10.3389/fchem.2022.1006389 (PMC9510749; doi:10.3389/fchem.2022.1006389)
Supplement: Supplementary file 1 [file DataSheet1.DOCX]

Supplementary Material

**Supplementary Table** **1.** The main deduced constituents present in collagen waste that shown in Figure 1b.

| *m*/*z* | Formula | Name |
| --- | --- | --- |
| 89 | C_3_H_7_NO_2_ | Alanine |
| 121 | C_3_H_7_NO_2_S | L-Cysteine |
| 133 | C_4_H_7_NO_4_ | Aspartic acid |
| 174 | C_6_H_14_N_4_O_2_ | Arginine |
| 274 | C_17_H_22_O_3_ | Fatty acid |
| 318 | C_18_H_22_O_5_ | Fatty acid |


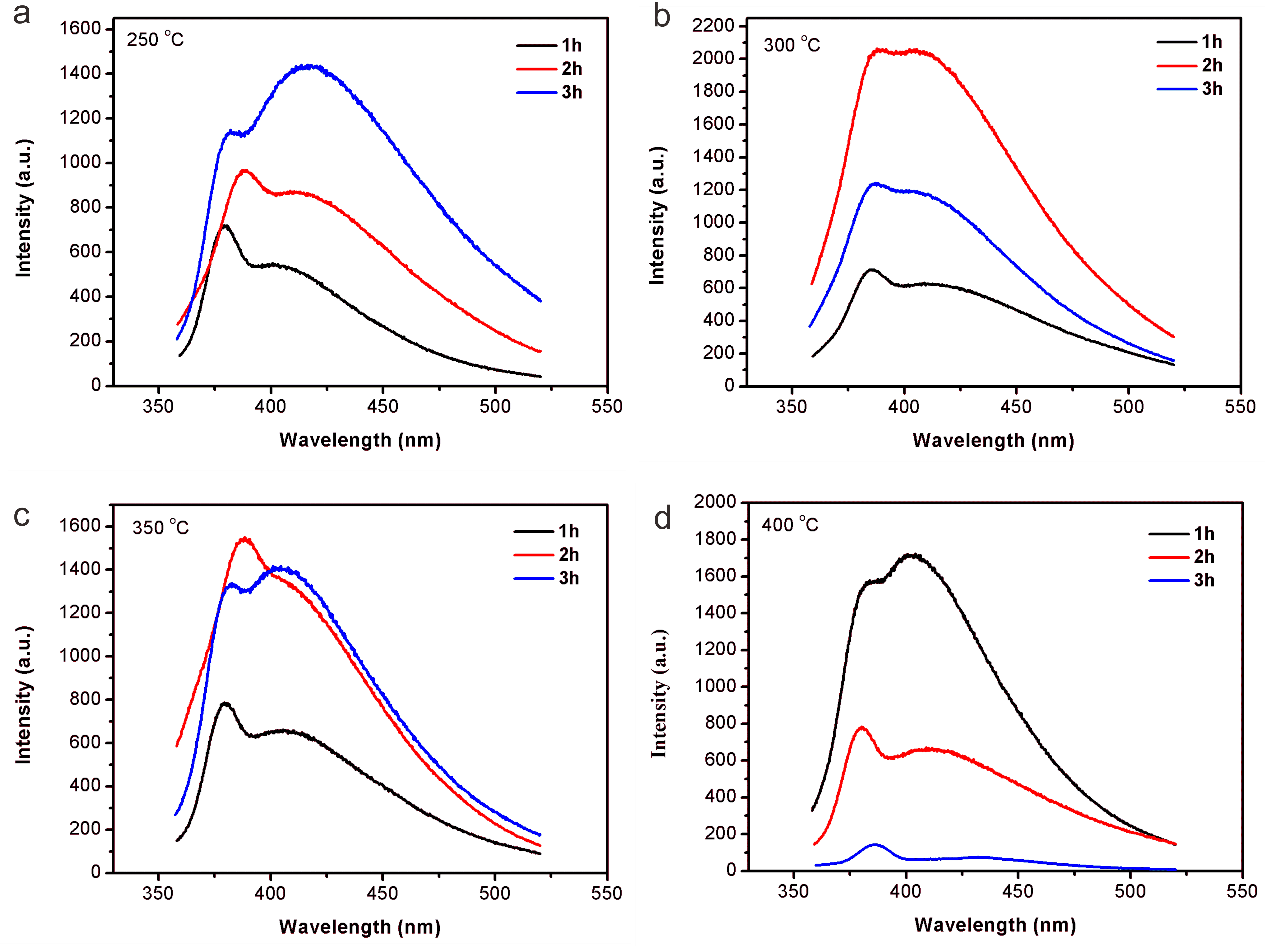


**Supplementary Figure 1.** The PL emission spectra of the aqueous dispersion of CDs (2.5 μg/mL) prepared under different calcining conditions.


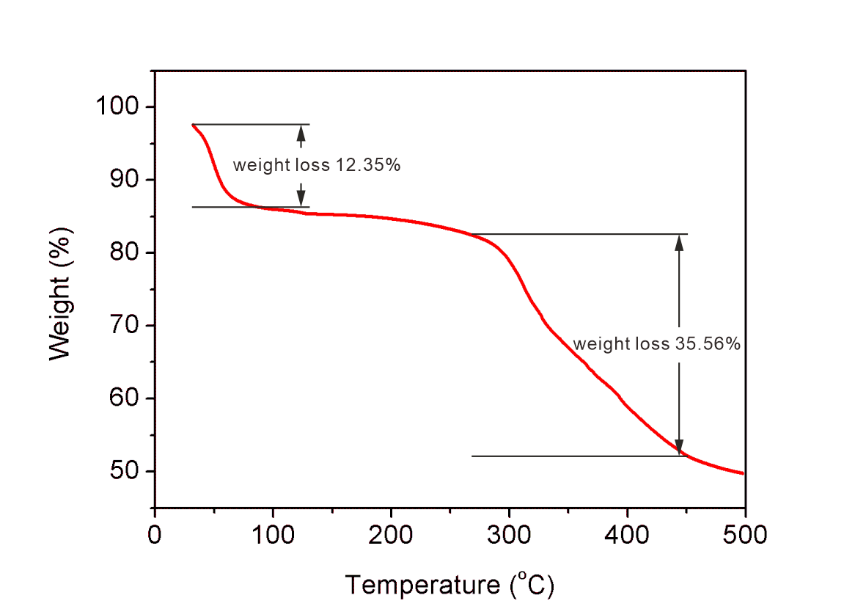


**Supplementary Figure 2.** The TGA spectra of the solid CDs. Temperature range: 30℃-500℃; Heating rate: 5℃/min; Gas: Nitrogen 100.0 mL/min.


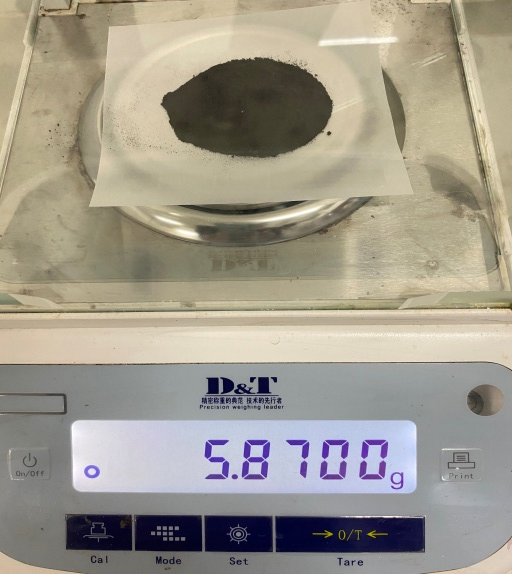


**Supplementary Figure 3.** The optical photograph of the product for one single preparation.





**Supplementary Figure 4.** The XRD pattern of solid CDs product.





**Supplementary Figure 5.** The excitation-dependent PL emission spectra of the aqueous dispersion of CDs (2.5 μg/mL).


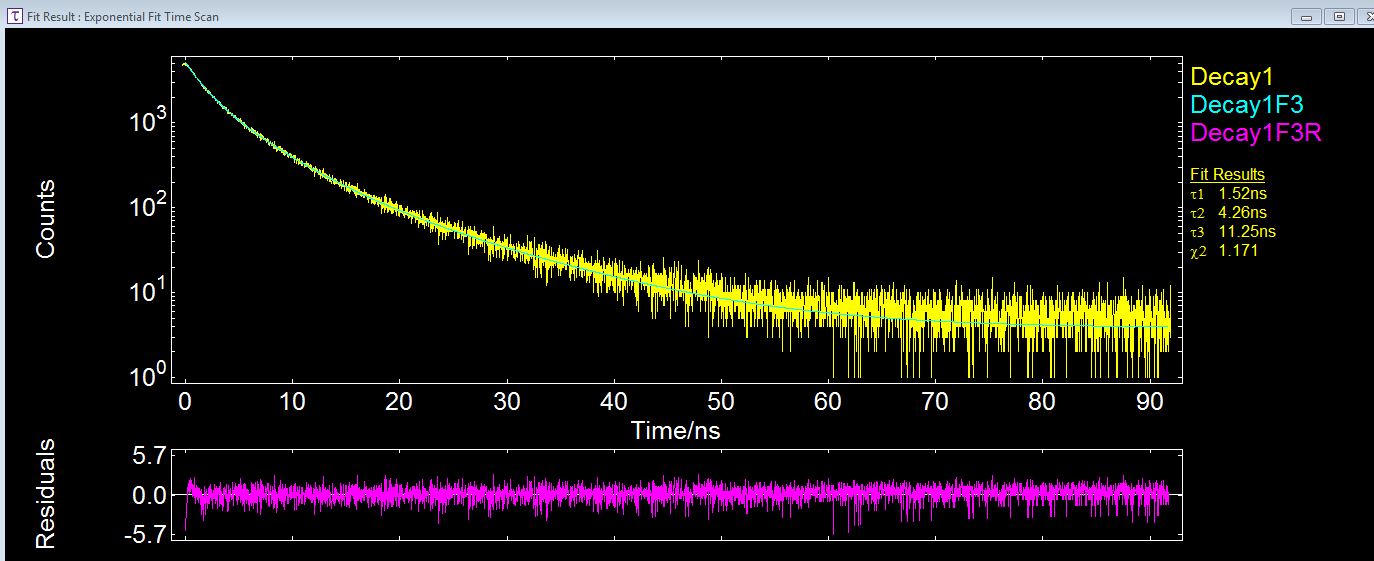


**Supplementary Figure 6.** The time-resolution photoluminescence measurements (λem: 413 nm, λex: 360 nm) of CDs.


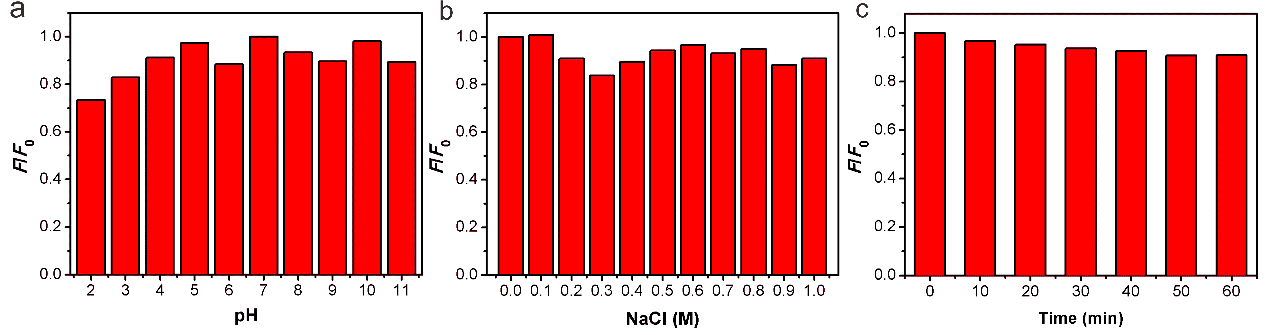


**Supplementary Figure 7.** The effect of (a) the solution pH value, (b) the NaCl concentration, and (c) UV illumination time on CDs fluorescence.


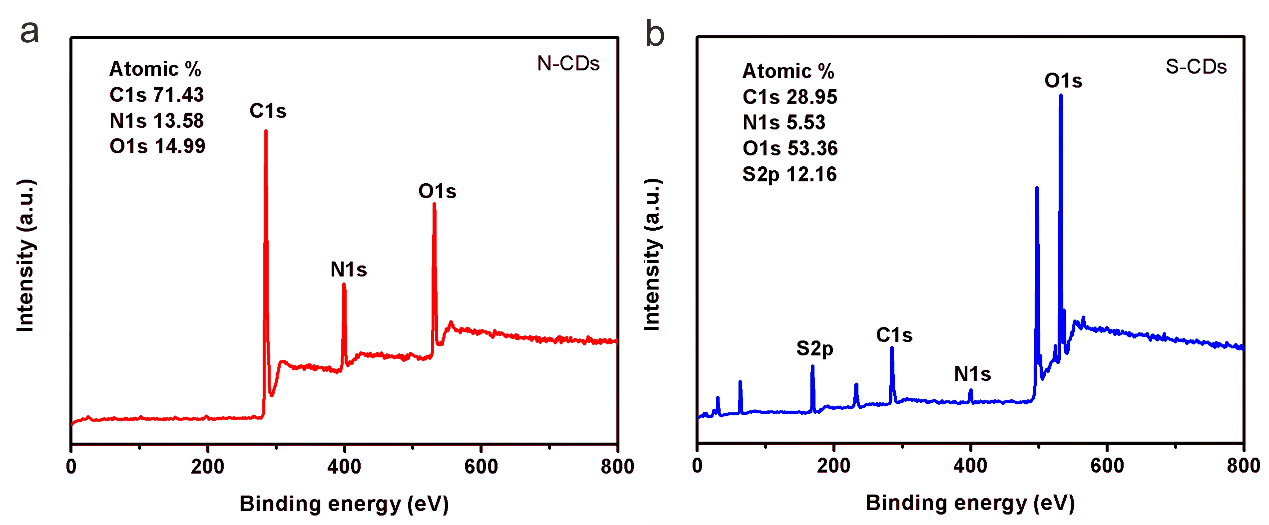


**Supplementary Figure 8.** The survey XPS spectra of (a) N-CDs and (b) S-CDs.





**Supplementary Figure 9.** The PL intensity of CDs in presence of different medium. All metal ions were at a concentration of 50 μM.


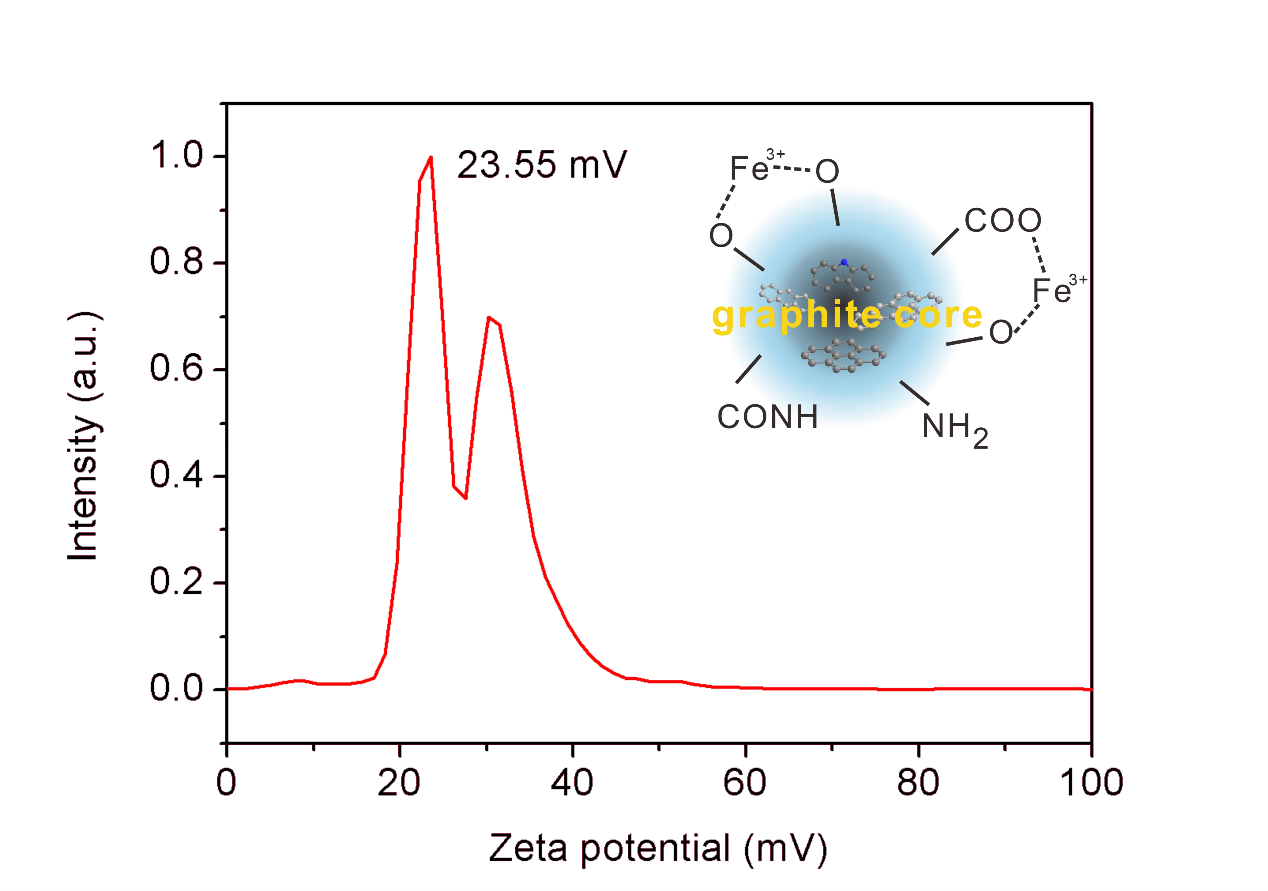


**Supplementary Figure 10.** The zeta potential of CDs aqueous solution accompanied with 100 μM Fe^3+^. The inset shows Fe^3+^ ions coordinate with the functional groups on the surface of CDs.





**Supplementary Figure 11.** The PL intensity attenuation curves of CDs in serum solution with increasing concentration of Fe^3+^.
